# Synergistic Effects of Polypropylene Fibers and Silica Fume on Structural Lightweight Concrete: Analysis of Workability, Thermal Conductivity, and Strength Properties

**DOI:** 10.3390/ma17205042

**Published:** 2024-10-15

**Authors:** Zehra Funda Akbulut, Eva Kuzielová, Taher A. Tawfik, Piotr Smarzewski, Soner Guler

**Affiliations:** 1 Department of Mining Engineering, Faculty of Engineering, University of Van Yüzüncü Yıl, Van 65080, Turkey; 2 Faculty of Chemical and Food Technology, Slovak University of Technology, 812 37 Bratislava, Slovakia; eva.kuzielova@stuba.sk; 3 Institute of Construction and Architecture, Slovak Academy of Sciences, 845 03 Bratislava, Slovakia; dr.taher_tawfik@csi.edu.eg; 4 Department of Construction and Building Engineering, High Institute of Engineering, October 6 City 12585, Egypt; 5 Faculty of Civil Engineering and Geodesy, Military University of Technology, 00-908 Warsaw, Poland; 6 Department of Civil Engineering, Faculty of Engineering, University of Van Yüzüncü Yıl, Van 65080, Turkey; gulersoner@yyu.edu.tr

**Keywords:** structural lightweight concrete, silica fume, polypropylene fibers, slump, Vebe time, thermal conductivity, compressive and splitting tensile strengths

## Abstract

Structural lightweight concrete (SLWC) is crucial for reducing building weight, reducing structural loads, and enhancing energy efficiency through lower thermal conductivity. This study explores the effects of incorporating silica fume (SF), micro-polypropylene (micro-PP), and macro-PP fibers on the workability, thermal properties, and strength of SLWC. SF was added to all mixtures, substituting 10% of the Portland cement (PC), except for the control mixture. Macro-PP fibers were introduced alone or in combination with micro-PP fibers at volumetric ratios of 0.3% and 0.6%. The study evaluated various parameters, including slump, Vebe time, density, water absorption (WA), ultrasonic pulse velocity (UPV), thermal conductivity coefficients (k), compressive strength (CS), and splitting tensile strength (STS) across six different SLWC formulations. The results indicate that while SF negatively impacted the workability of SLWC mortars, it improved CS and STS due to the formation of calcium silicate hydrate (C-S-H) gels from SF’s high pozzolanic activity. Additionally, using micro-PP fibers in combination with macro-PP fibers rather than solely macro-PP fibers enhanced the workability, CS, and STS of the SLWC samples. Although SF had a minor effect on reducing thermal conductivity, the use of macro-PP fibers alone was more effective for improving thermal properties by creating a more porous structure compared to the hybrid use of micro-PP fibers. Moreover, increasing the ratio of micro- and macro-PP fibers from 0.3% to 0.6% resulted in lower CS values but a significant increase in STS values.

## 1. Introduction

Structural lightweight concrete (SLWC) has recently emerged as a favored construction material in the building sector due to its technical, economic, and environmental advantages. This type of concrete reduces the self-weight of structures and minimizes the cross-sectional areas of reinforced concrete elements, enhancing architectural flexibility, especially in high-rise buildings [1]. It finds wide application in various scenarios, such as wall panels, blocks, roof slabs, bridge spans, and precast concrete units, and it is frequently preferred in seismic zones [2]. The most common method employed in lightweight concrete production is using porous lightweight aggregates, which significantly influence its mechanical properties and other characteristics. Some researchers have reported that lightweight aggregates contribute to a higher strength-to-weight ratio, possess low thermal expansion coefficients, and offer superior thermal and sound insulation properties [3]. In Turkey, lightweight concrete production commonly involves natural or artificial porous aggregates, with extensively utilized materials like pumice, volcanic tuff, and volcanic scoria [4]. Pumice stone, abundant in the central and eastern Anatolian regions of Turkey, is a natural lightweight aggregate with a porous structure and low unit volume weight, making it suitable for insulation concrete and SLWC production [5]. Karakurt and Özen [6] highlight that Turkey’s abundant natural lightweight aggregates, particularly pumice and volcanic tuffs, are extensively utilized in the production of lightweight concrete. These materials provide significant advantages in both insulation and structural applications. Sang [7] explains that although concrete composed of pumice aggregate (PA) has generally been deemed inadequate for load-bearing purposes, its efficacy can be enhanced with the incorporation of ingredients such crushed gravel, fly ash, and limestone powder. Yeğinobali et al. [8] further demonstrate that high-strength lightweight concrete can be produced using natural aggregates like volcanic tuff and pumice combined with silica fume and superplasticizers. This method can achieve compressive strengths of up to 55 MPa, dry unit weights ranging from 1700 to 2100 kg/m^3^, and thermal conductivity coefficients around 0.55 W/mK. Overall, these natural lightweight aggregates present cost-effective alternatives to artificial aggregates and hold promise for creating structural lightweight concrete with enhanced strength and insulation properties.

Sustainable development emphasizes the necessity of preserving environmental resources as finite commodities while ensuring effective waste management [9]. The increasing global waste, estimated at around 2 billion tons annually, has prompted the need for innovative disposal methods [10]. In the cement manufacturing sector, there are numerous opportunities to utilize leftover raw materials in concrete production [11]. Waste materials can serve as substitutes for cement or as fine and coarse aggregates in concrete mixtures. Concrete stands as the most widely used man-made construction material in the construction industry, with hydraulic cement being a critical component [12]. In 2015 alone, approximately 20–30 billion tons of concrete were produced, requiring vast amounts of hydraulic cement each year [13]. Reports from the World Cement Association Conference indicate a steady rise in global cement production rates [14]. This growth can be largely attributed to the increasing demand for cement in modern construction and infrastructure, particularly in developing nations such as China, Turkey, and Japan [15]. Consequently, it is essential to explore alternative materials wherever possible to reduce reliance on traditional cement [16]. Utilizing alternative materials not only mitigates the pressure on cement production but also contributes to environmental sustainability. By integrating recycled waste into concrete, the construction industry can decrease landfill contributions and lower carbon emissions associated with cement manufacturing. Furthermore, leveraging by-products from other industries can enhance the mechanical properties of concrete, creating a dual benefit of resource efficiency and improved material performance. As the construction sector evolves, prioritizing sustainable practices becomes imperative for balancing economic growth with environmental stewardship [17].

In recent years, the incorporation of various types of pozzolanic materials into SLWC mixtures through the partial replacement of cement has facilitated the production of more environmentally friendly, higher strength, and durable concrete [18]. One notable pozzolanic material is silica fume (SF), a finely divided, non-crystalline silica generated as a by-product in the production of silicon or silicon-containing alloys in electric arc furnaces. SF acts as both a mineral and pozzolanic admixture in concrete, functioning at the micro-scale to significantly enhance the material properties of the concrete mixture. The addition of SF effectively improves the granulometry of the concrete mixture by filling the voids between cement particles, thus optimizing the packing density and contributing to a more compact microstructure [19]. This improved microstructure is crucial for enhancing the mechanical properties of the concrete. Furthermore, the pozzolanic reaction of SF with calcium hydroxide (CH), a by-product of the cement hydration process, results in the formation of calcium silicate hydrate (C-S-H) gel, which plays a pivotal role in the strength development of concrete, as it contributes substantially to the binding and structural integrity of the material. However, it is important to recognize that the high surface area of SF increases the water demand in the concrete mixture. This necessitates careful consideration of the water-to-cement ratio to achieve the desired workability and strength characteristics without compromising the overall performance of the SLWC. Addressing these challenges through meticulous mixture design and the incorporation of appropriate superplasticizers can further optimize the effectiveness of SF in SLWC applications, ultimately resulting in a superior composite material characterized by enhanced mechanical and durability properties [20].

Furthermore, the incorporation of various types of steel and synthetic fibers into SLWC mixtures can significantly enhance strength, ductility, and durability properties. In recent years, among the most commonly used synthetic fibers for SLWC production are polyester, polyolefin, polyethylene, polyvinyl alcohol, and polypropylene (PP) fibers [21]. PP fibers, specifically, are extensively utilized in lightweight concrete mixtures and traditional concrete applications such as pavements, industrial flooring, airport runway coatings, water structures, shotcrete, and slopes that require stability [22]. These fibers are characterized by their excellent tensile strength and flexibility, which allow them to effectively distribute stresses throughout the concrete matrix [23]. One of the critical advantages of PP fibers is their effectiveness in limiting plastic shrinkage and cracking [24]. During the early curing stages, concrete is particularly susceptible to cracking due to shrinkage; the addition of PP fibers helps mitigate this issue by creating a three-dimensional reinforcement network within the matrix. Additionally, PP fibers enhance the concrete’s capacity to undergo deformation, increase its toughness, and improve both tensile and flexural strengths, thereby providing superior resistance against impacts and fire. Moreover, the lower unit volume weight of PP fibers, combined with their corrosion resistance compared to steel fibers, makes them an attractive option for use in both traditional and SLWC mixtures. Their ability to maintain performance over time, even in challenging environments, further underscores their value as a reinforcement material in sustainable concrete applications [25].

## 2. The Novelty of This Study

SLWC plays a critical role in reducing the weight of modern structures, alleviating structural loads, and enhancing energy efficiency. In this context, numerous researchers have explored the effects of various pozzolanic materials, such as fly ash, silica fume, blast furnace slag, and metakaolin, as well as different types of synthetic and steel fiber additives on SLWC. However, there are significant variations among these studies regarding the parameters used, including SLWC mixture components, types of cement, proportions of pozzolanic materials, and types and volumetric ratios of fibers. Currently, there is a gap in the literature regarding the comprehensive examination of the SLWC specimens incorporating both SF and micro- and macro-PP fibers simultaneously with respect to workability, physical, mechanical, and thermal properties. This study aims to fill this void and provide an innovative contribution to the field. Initially, we investigate the impact of SF on the workability, thermal, physical, and strength properties of SLWC. Following this, the effects of the combined use of SF with micro- and macro-PP fibers on SLWC properties will be evaluated. Notably, the comparison of hybrid mixtures using macro-PP fibers alongside micro-PP fibers with those containing only macro-PP fibers represents a significant and unique aspect of this research.

Moreover, the sustainable benefits of using both SF and PP fibers together are expected to contribute meaningfully to the existing literature. Ultimately, this research will serve as an important resource for the development of SLWC formulations and the construction of more environmentally friendly and durable structures in engineering applications. In this regard, our study is anticipated to create profound impacts both theoretically and practically.

To this end, an attempt was made to determine to what extent the physical, thermal, and mechanical properties of SLWC mixtures are affected by the inclusion of SF as a waste pozzolanic material along with micro-PP fibers measuring 12 mm in length and macro-PP fibers measuring 40 mm in length. Initially, fresh properties such as slump, Vebe time, DD, and ODD of SLWC mixtures were investigated. Then, WA, UPV, k, CS, and STS of the SLWC specimens were evaluated. In the final stage of the study, microstructural images of selected SLWC specimens were obtained and evaluated using scanning electron microscopy (SEM) analysis. This comprehensive approach aims to provide insights into the influence of micro- and macro-PP fibers and SF pozzolanic additives on the properties of SLWC mixtures, thereby contributing to the development of more advanced and sustainable construction materials.

## 3. Experimental Program

### 3.1. Materials Used in the SCLWC Mixtures

**Figure 1:**
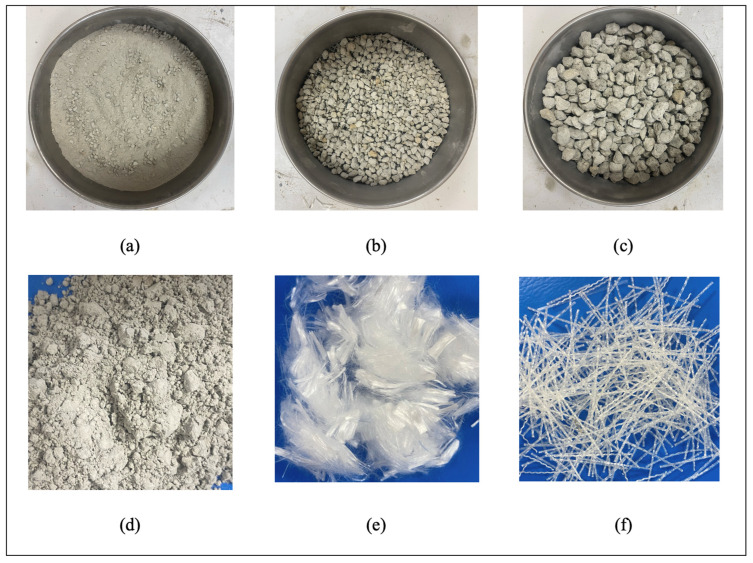
The images of the PA_s_ ((**a**) 0–4 mm, (**b**) 4–8 mm, and (**c**) 8–16 mm), (**d**) SF, (**e**) 12 mm micro-PP, and (**f**) 40 mm macro-PP fibers.

**Figure 2:**
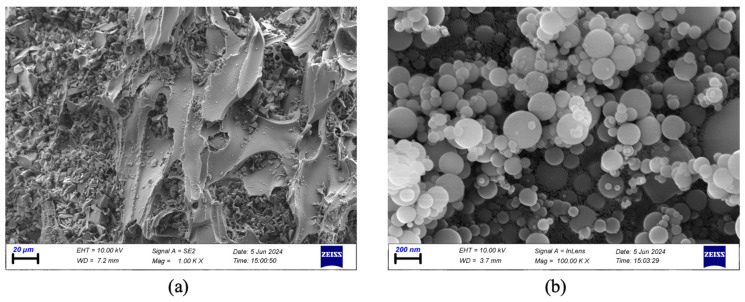
The SEM images of the pumice aggregate and silica fume (SF). (**a**) pumice aggregate (**b**) silica fume.

In preparing SLWC mixtures, CEM I 42.5 R Portland Cement (PC) produced by TS EN 197-1 [26] standard by Van Cement Factory was used. For SLWC mixtures, PA with particle sizes of 0–4 mm, 4–8 mm, and 8–16 mm were used as fine aggregates. SF was used as pozzolanic material. SF is a waste material produced by ferrochrome plants and is available for purchase online. In the SLWC mixtures, macro-PP fibers with a length of 40 mm are used in a single form, and micro-PP fibers with a length of 12 mm are used in a hybrid form. Micro-PP and macro-PP fibers are used in SLWC mixtures at 0.3% and 0.6% volumetric ratios, respectively. A superplasticizer (SP) admixture was used to ensure suitable workability in all mixtures. To achieve appropriate workability and to make meaningful comparisons in fresh concrete properties, the SP ratio was taken as 1% of the binder by weight in all mixtures. Images of pumice aggregate (0–4 mm, 4–8 mm, and 8–16 mm), SF, 12 mm micro-PP, and 40 mm macro-PP fibers are presented in Figure 1. The interior images of pumice aggregate and SF were obtained with a scanning electron microscope (SEM) analysis under ambient conditions, as shown in Figure 2. The chemical and physical properties of pumice aggregates is given in Table 1 and Table 2, respectively. The technical properties of PC and SF are given in Table 3. Table 4 presents the technical specifications of micro- and macro-PP fibers. The technical specifications for micro- and macro-PP fibers were obtained from the manufacturer. The chemical composition of PA, PC, and SF was ascertained using X-ray fluorescence (XRF) analysis, with all results expressed as weight percentages (wt%). Table 1 presents the XRF results for pumice aggregates (PA_s_), whereas Table 3 delineates the results for PC and SF. Air-dried PA samples were placed into a calibrated measuring cup to ascertain the loose bulk density in accordance with TS EN 1097-3 [27]. Precautions were implemented to avert the segregation of the aggregate and harm to the coated surfaces, filling the cup to the point of overflow. The upper surface of the filled sample cup was manually leveled to prevent compression. The weight of the filled sample cup was then documented, and the results were computed. The WA test for the PA_s_ was conducted in accordance with the TS EN 1097-6 [28] standard. PA_s_ were placed in an oven at 105 °C until they reached a constant weight, after which a sample was weighed. A portion of the PA_s_, dried in the oven, was immersed in water for 24 h. After this period, excess water was removed with a dry cloth to achieve a saturated surface-dry condition, and the weights were measured to determine the WA rates.

### 3.2. Mixing Phases of SCLWC Mixtures

The mixture design of the SLWC was conducted based on the absolute volume method according to the TS EN 206-1 [29] and TS 2511 [30] standards. In the SLWC mixtures, the binder dosage was kept constant at 400 kg/m^3^ and the water/cement ratio was maintained at 0.52, while the amounts of other materials used in the mixtures were determined accordingly. A concrete mixer was utilized to prepare the SLWC mixtures. Initially, the K0 and K1 mixtures comprised a dry blend of PC, PA, and SF in the concrete mixer for a duration of two minutes. Subsequently, the total requisite water and SP ingredient were incorporated and the mixture was blended for three minutes. Micro- and macro-PP fibers were included into the K2–K5 mixtures. Initially, PC, PA, and SF were dry-mixed for two minutes. Subsequently, fifty percent of the necessary water was included into the mixture. Subsequent to the manual addition of micro- and macro-PP fibers, the residual water and superplasticizer (SP) were integrated, and the mixture was blended for three minutes. After mixing, initial slump, Vebe time, DD and ODD were obtained for SLWC mixtures. Then, WA, UPV, k, CS, and STS of hardened SLWC samples were determined. The CS, STS, and k values of the SLWC samples were determined using cube specimens with a side length of 150 mm. The produced SLWC samples were kept in molds in ambient conditions for 24 h. At the end of this period, the samples were removed from the molds and cured in a curing tank in water at a temperature of 20 ± 2 °C for 28 days. In all experiments, three samples were tested for each mixture, and the average was taken. The material quantities used in SLWC mixtures are given in Table 5.

### 3.3. Tests of SLWC Samples

**Figure 3:**
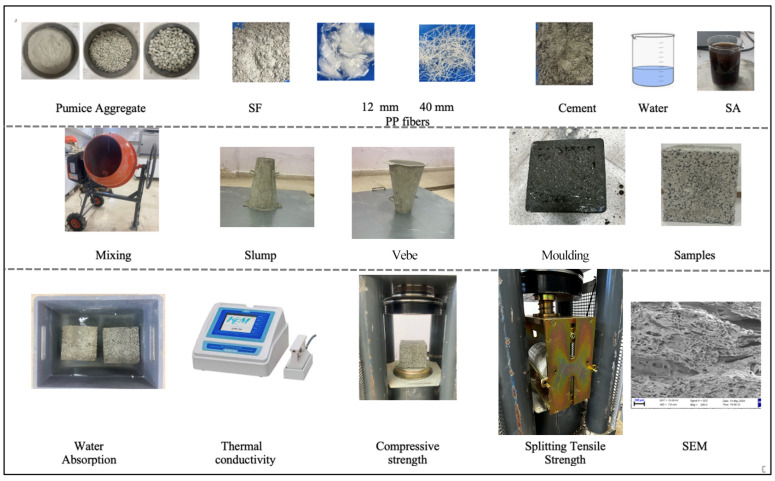
A schematic overview of the experimental procedure.

Fresh SLWC mixture slump values were determined according to the TS EN 12350-2 standard [31], while Vebe time values were calculated by the TS EN 12350-3 standard [32]. SLWC specimens are subjected to WA tests to evaluate their porosity and permeability properties. Typically conducted according to standards such as ASTM C1585 [33], the test involves immersing the specimens in water or applying water pressure to their surfaces. After a specified period, the specimens are removed, and their mass gain due to water absorption is measured to assess the concrete’s resistance to moisture ingress. UPV test for SLWC samples is conducted to evaluate its microstructure and integrity through the propagation of ultrasonic waves. Typically performed by standards such as ASTM C597 [34], the procedure involves placing pairs of transducers on the surface of a concrete SLWC specimen. Heat conductivity measurements were performed on SLWC cube specimens with dimensions of 150 mm × 150 mm × 150 mm. The KEM QTM-500 test device (KEM, Kyoto, Japan) was utilized for insulation tests with a measurement range of 0.023–11.63 W/mK. Due to the inability of the experimental device to perform measurements on moist materials, the measurements were conducted on specimens dried in an oven following the EN 1745 standard [35]. The obtained results were taken as the arithmetic average of the three experiments. The CS and STS of SLWC specimens at 7 and 28 days were determined by TS EN 12390-3 [36] and TS EN 12390-6 [37] standards, respectively. SEM observations of K0, K1, and K5 samples were taken from the analysis of SLWC samples broken into very small sizes under 1 × 200 and 1 × 1000 magnifications. In the study, cube samples 150 mm in side were used for WA, UPV, k, and CS tests. For the STS test, cylindrical sample molds measuring 150 × 300 mm were utilized. The CS and STS values of SLWC specimens were calculated by taking the arithmetic average of three specimens broken for each experiment. The schematic representation of the preparation and testing stages of SLWC specimens is provided in Figure 3.

## 4. Results and Discussion

### 4.1. Slump, Vebe Time, De-Molded Density, and Oven-Dry Density of SLWC Mixtures

**Figure 4:**
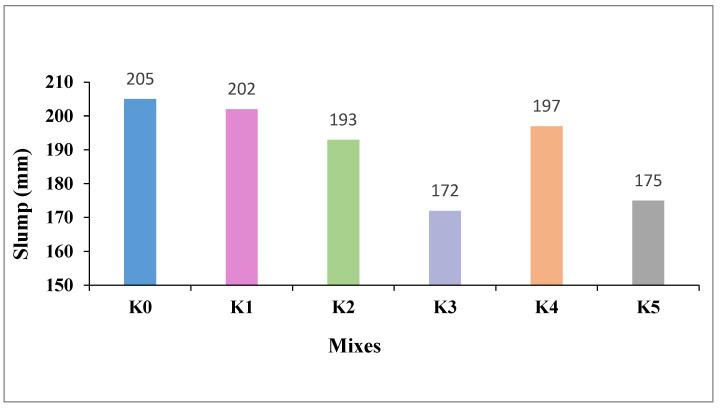
The slump values of fresh SLWC mixtures.

The slump values and Vebe times of the fresh SLWC mixtures denoted as K0–K5, are provided in Figure 4.

**Figure 5:**
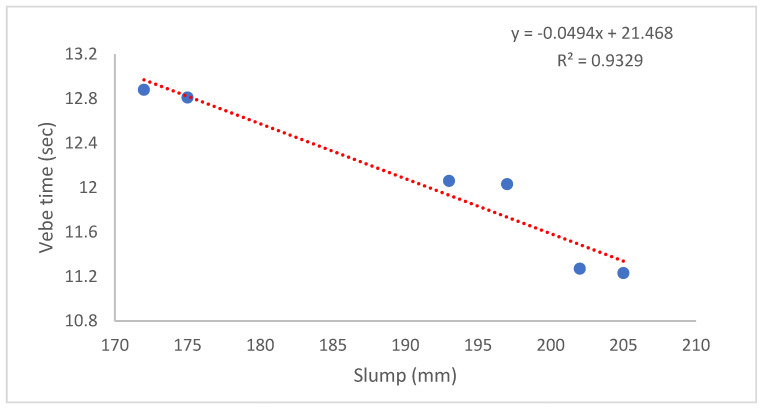
The Vebe time–slump relationship of fresh SLWC mixtures.

As seen in Figure 4, the slump value of the control mixture (K0) is equal to 205 mm. Similarly, the slump value of the mixture with only the SF additive (K1) is also 202 mm. The mixtures with 0.3% and 0.6% macro-PP fiber additions (K2 and K3) exhibit slump values of 193 and 172 mm, respectively. The slump values of the mixtures (K4 and K5), where micro-PP fibers are used in hybrid form alongside 0.3% and 0.6% macro-PP fibers, respectively, are also 197 and 175 mm. Moreover, Vebe times for the K0, K1, K2, K3, K4, and K5 SLWC mixtures are obtained as 11.23, 11.27, 12.06, 12.88, 12.03, and 12.81 s, respectively. The graph showing the relationship between Vebe time and slump is given in Figure 5. These results show that both SF and PP fibers adversely affect the workability of fresh SLWC mixtures, decreasing slump values and increasing Vebe times. The primary reason for SF’s reduction in workability is attributed to its high specific surface area (SSA), causing it to adsorb mixing water, thereby reducing the flowability and workability of the mixture [38]. Chandra and Hardjito [39] investigated the effects of using fly ash (0–30%), SF (0–10%), and calcium carbonate (0–15%) as partial replacements for cement. Their findings indicate that increasing fly ash enhances both the workability and compressive strength of the mortar, while SF reduces workability, necessitating the addition of a superplasticizer for improvement. Overall, while fly ash increases flow diameter, a higher SF content increases superplasticizer demand to maintain the desired flow.

Incorporating micro- and macro-PP fibers into the mixtures reduces their flowability and slump values, consequently increasing the Vebe times. This is primarily due to the tendency of PP fibers to agglomerate within the mixture, which significantly reduces the workability of the blend. However, utilizing micro-PP fibers alongside 40 mm long macro-PP fibers slightly improves the workability of the mixtures, resulting in marginally higher slump values. Using micro-PP fibers in hybrid form instead of solely macro-PP fibers mitigates the larger frictional forces between macro-PP fibers, consequently reducing the plastic viscosity of the mixture, making it more fluid and achieving higher slump values. Hosseinzadeh et al. [40] noted that micro- and macro-PP fibers can create a network structure in fresh concrete, making it more difficult for coarse aggregates to move relative to each other, which ultimately reduces the mobility of the mixture. In contrast, basalt (BA) fibers have a minimal impact on the movement of coarse aggregates due to their lower flexural strength. However, BA fibers can adsorb more water than macro-PP fibers because of their higher specific surface area (SSA), which can result in lower workability. Heo et al. [41] found that incorporating long PP fibers leads to a decrease in the workability of concrete. Mohod [42] further explained that the dimensions of PP fibers, along with the specific mixture design, prevent the fibers from clumping together. As a result, the addition of PP fibers can significantly reduce the volume drop, achieving a decrease of approximately 0.1%. This suggests that while long PP fibers can negatively impact workability, careful mixture design can mitigate the extent of this effect.

**Figure 6:**
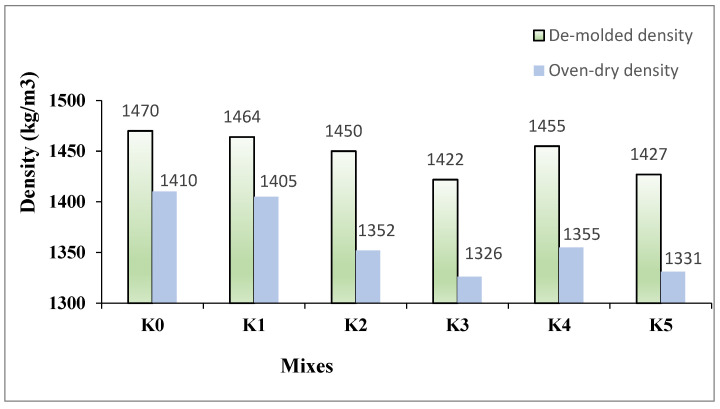
The de-molded density (DD) and oven-dry density (ODD) values of K0–K5 SLWC mixtures.

Furthermore, this phenomenon leads to slightly longer Vebe times for the hybrid K4–K5 SLWC mixtures than the K2–K3 mixtures using only macro-PP fibers. The DD and ODD values of the K0–K5 SLWC mixtures are presented in Figure 6. As depicted in Figure 6, compared to the K0 control mixture, the finer particle size of SF in the K1 specimens slightly decreases both the DD and ODD values of the SLWC specimens. In addition, adding micro- and macro-PP fibers into the K2–K5 mixtures results in lower DD and ODD values than the K0 control mixture. One of the main reasons for this is the voids created by incorporating micro- and macro-PP fibers within the SLWC matrix. However, compared to the single macro-PP fiber usage, the hybrid PP fiber incorporation fills the voids slightly better within the matrix, resulting in marginally higher DD and ODD values

### 4.2. Water Absorption of SLWC Specimens

**Figure 7:**
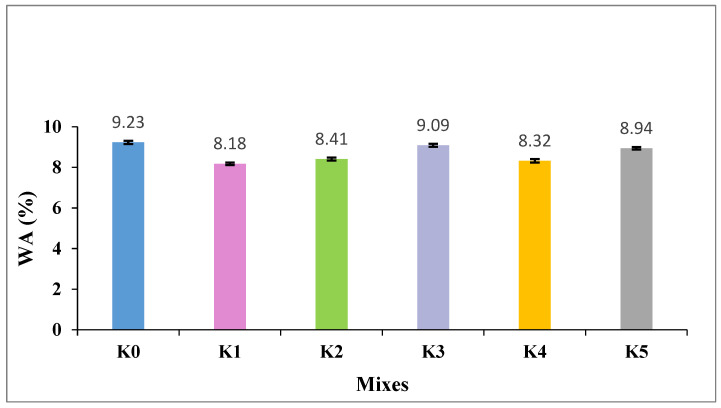
The water absorption (WA) values of SLWC specimens.

The WA values of the SLWC specimens K0–K5 are provided in Figure 7.

As observed in Figure 7, the WA value of the control mixture (K0) is 9.23% and the WA value of the mixture with only the SF additive (K1) is 8.18%. The mixtures with 0.3% and 0.6% macro-PP fiber additions (K2 and K3) exhibit WA values of 8.41% and 9.09%, respectively. Additionally, the WA values of the mixtures (K4 and K5), where micro-PP fibers are used in hybrid form alongside 0.3% and 0.6% macro-PP fibers, respectively, are 8.32% and 8.94%. The WA values of the SLWC specimens, particularly concerning the influence of SF and PP fibers, are crucial in understanding the performance characteristics of these materials. SF plays a significant role in reducing water absorption in SLWC mixtures. Its fine particle size and pozzolanic properties enable it to pack densely within the matrix, filling voids and pore spaces. This results in a more impermeable microstructure, decreasing water absorption. However, while SF contributes to improved water resistance, its high surface area may also increase water demand during mixing, affecting workability. Naik et al. [43] highlight that SF and fly ash, both industrial solid wastes, can be effectively utilized in concrete to enhance its properties while addressing environmental concerns. Yuvraj et al. [44] explain that these supplementary cementitious materials significantly reduce WA and capillary action by promoting a more homogeneous and compact cement matrix. They also note that the inclusion of nanoparticles, such as nanosilica, improves water absorption resistance by filling pores and reacting with calcium hydroxide (CH) crystals. Furthermore, Wittmann et al. [45] demonstrate that polymers like silicon resins can increase the wetting angle of pore walls in cement-based materials, effectively preventing capillary suction and minimizing water uptake. Overall, these modifications enhance concrete durability and extend the service life of reinforced concrete structures in aggressive environments. Ghewa et al. [46] report that incorporating 15% fly ash into normal concrete enhances water penetration resistance by 23%, whereas 5% SF results in an 11.8% increase. Additionally, Hall [47] describes sorptivity as a material property that quantifies the tendency of porous materials to absorb and transmit water through capillarity, effectively measuring the WA characteristics of concrete.

On the other hand, incorporating PP fibers introduces a contrasting effect on water absorption. Due to their relatively larger size, macro-PP fibers may create voids within the matrix, consequently increasing water absorption. Additionally, the hydrophobic nature of PP fibers may repel water, resulting in reduced interfacial bonding between the fibers and the cementitious matrix, further exacerbating water ingress. In contrast, micro-PP fibers, primarily used in hybrid form alongside macro-PP fibers, can enhance the overall performance of SLWC mixtures regarding water absorption. The finer size and improved dispersion of micro-PP fibers allow them to fill the voids created by macro-PP fibers more effectively, leading to a denser microstructure with reduced WA values of the SLWC specimens.

Eidan et al. [48] demonstrate that PP fibers significantly enhance various properties of concrete, with 6 mm fibers increasing energy absorption capacity by 69% and 12 mm fibers reducing water permeability by 53%. Zia and Ali [49] note that concrete containing 5% PP fibers by mass of cement exhibits lower WA and linear shrinkage compared to plain concrete. Sadrmomtazi and Fasihi [50] highlight that incorporating PP fibers into cementitious composites can yield complex effects on WA and mechanical properties. While these fibers may create voids that increase WA, their hydrophobic nature can also repel water, potentially enhancing waterproofing performance. The fiber–matrix interface is crucial for determining the overall properties of the composite. Rostami et al. [51] explain that surface modifications of PP fibers, such as indentation or chemical treatments to enhance hydrophilicity, can improve fiber–matrix adhesion and boost mechanical properties, including flexural strength and energy absorption capacity.

### 4.3. Ultrasonic Pulse Velocity of SLWC Specimens

**Figure 8:**
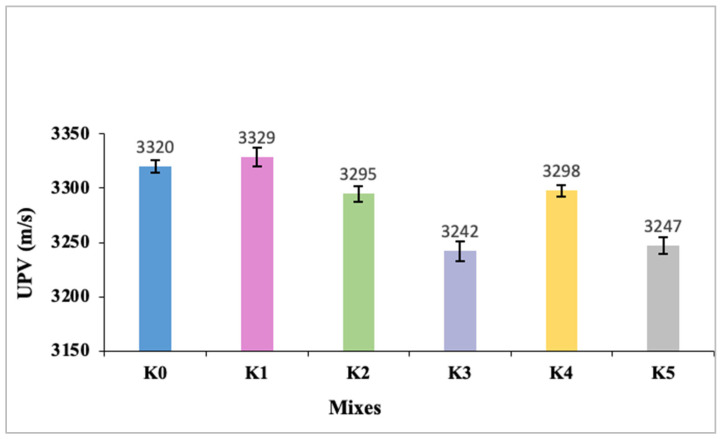
The ultrasonic pulse velocity (UPV) values of SLWC specimens.

The UPV values of the SLWC specimens K0–K5 are presented in Figure 8. As depicted in Figure 8, the UPV value of the control mixture (K0) is 3320 m/s and the UPV value of the mixture with the SF additive (K1) is 3329 m/s. The mixtures with 0.3% and 0.6% macro-PP fiber additions (K2 and K3) exhibit UPV values of 3295 m/s and 3242 m/s, respectively.

Additionally, the UPV values of the mixtures (K4 and K5) where micro-PP fibers are used in hybrid form alongside 0.3% and 0.6% macro-PP fibers, respectively, are 3298 m/s and 3247 m/s, respectively. The primary reason for SF’s reduction in UPV values in the mixtures is attributed to its filler effect, effectively filling voids within the SLWC matrix and resulting in a denser microstructure. Rodríguez et al. [52] and Sikora et al. [53] highlight that the incorporation of SF and nanosilica (NS) into cement-based materials significantly impacts their microstructure and properties. Both SF and NS enhance compressive strength and refine pore structure through pozzolanic reactions and filler effects. Notably, Sikora et al. [53] indicate that NS demonstrates higher efficiency than SF in improving the mechanical and transport properties of lightweight concretes. Additionally, Hong et al. [54] discuss how the addition of these materials alters hydration kinetics and microstructures, resulting in varying strengths and UPV depending on the replacement rate. Zheng et al. [55] further explain that nanosilica fume (NSF) in lightweight cement slurry improves compressive strength and microstructure through pozzolanic and nucleation effects, consuming portlandite to form more calcium silicate hydrate and providing nucleation sites for cement hydration. However, incorporating micro- and macro-PP fibers into the mixtures creates voids within the SLWC specimens’ matrix, consequently decreasing UPV values. Nevertheless, utilizing micro-PP fibers alongside 40 mm long macro-PP fibers effectively fills the micro-voids within the SLWC specimens’ matrix, resulting in slightly lower UPV values for the mixtures. This observation underscores the contrasting effects of SF and micro- and macro-PP fibers on the microstructure and ultrasonic velocities of SLWC. While SF enhances densification and increases UPV values through its filler effect, PP fibers introduce voids that lead to a reduction in UPV values. However, combining micro- and macro-PP fibers allows for a more optimized microstructure, enhancing mechanical properties and slightly lower UPV values in SLWC mixtures. Hedjazi and Castillo [56] discuss the varying effects of incorporating PP fibers into concrete mixtures. While these fibers can enhance durability and electrical resistivity, they may slightly decrease CS and UPV. However, Alyousef et al. [57] also note that PP fibers significantly improve tensile strength and sound insulation, especially at higher dosages. Hedjazi and Castillo [56] depicted that the addition of PP fibers alters UPV propagation, leading to the necessity of developing new equations to accurately predict compressive strength based on UPV values. Shoba and Asha [58] found optimal results with 20% microsilica and 2% PP fibers, which increased both UPV and CS. Additionally, Chajec et al. [59] report that PP fibers affect the properties of concrete mixtures, causing changes in consistency and increasing air content without significantly altering bulk density. These findings highlight the complex interactions between PP fibers and concrete, emphasizing the need for careful consideration when incorporating them into mixtures.

### 4.4. Thermal Conductivity of SLWC Specimens

**Figure 9:**
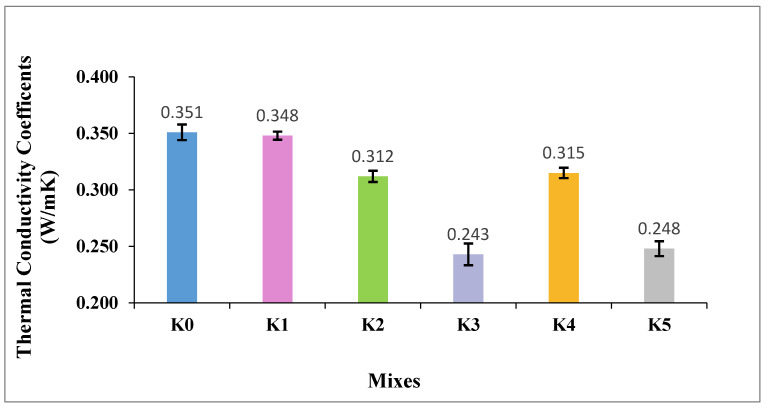
The thermal conductivity coefficient (k) values of SLWC specimens.

The k values of the SLWC specimens (K0–K5) are provided in Figure 9.

As observed in Figure 9, the k of the control mixture (K0) is 0.351 W/mK and the k of the mixture with the SF additive (K1) is 0.348 W/mK. The mixtures with 0.3% and 0.6% macro-PP fiber additions (K2 and K3) exhibit thermal conductivity values of 0.312 W/mK and 0.243 W/mK, respectively. Additionally, the k values of the mixtures (K4 and K5), where micro-PP fibers are used in the hybrid form alongside 0.3% and 0.6% macro-PP fibers, respectively, are also 0.315 W/mK and 0.248 W/mK, respectively. From these results, SF slightly reduces the k values of the SLWC samples. One of the most important reasons for this situation is that it somewhat reduces the density values of the SLWC samples due to having a slightly lower density value than PC. In addition, incorporating micro- and macro-PP fibers into the mixtures decreases k values. Adding PP fibers increases porosity within the SLWC specimens due to their hydrophobic nature, leading to water retention during curing. As this water is later evaporated, additional voids are left behind within the matrix. As previously mentioned, these voids trap heat and slow its transfer, resulting in lower thermal conductivity. Including PP fibers in SLWC mixtures increases the overall porosity percentage, thereby reducing thermal conductivity. Moreover, using macro-PP fibers alone in the specimens K2–K3 creates larger voids within the matrix compared to the hybrid utilization of micro-PP fibers alongside macro-PP fibers in the mixtures K4–K5. This difference in void size contributes to slightly lower thermal conductivity values in the K2–K3 specimens compared to the K4–K5 mixtures. Poonyakan et al. [60] and Jhatial et al. [61] demonstrate that the incorporation of PP fibers into concrete mixtures increases overall porosity and reduces thermal conductivity. Jhatial et al. [61] further show that adding PP fibers to lightweight foamed concrete decreases thermal conductivity while simultaneously improving mechanical properties. Similarly, Ahmed et al. [62] found that using waste PP as a partial replacement for sand in concrete mixtures led to a 49.1% reduction in thermal conductivity at a 40% PP content. Additionally, Amaral Jr and Moravia [63] indicate that the addition of PP fibers alters other thermal properties of concrete, such as reducing thermal expansion. These findings suggest that incorporating PP fibers can effectively enhance the thermal insulation properties of concrete; however, careful consideration of the optimal fiber content is essential to balance thermal and mechanical performance.

### 4.5. Compressive Strength of SLWC Specimens

**Figure 10:**
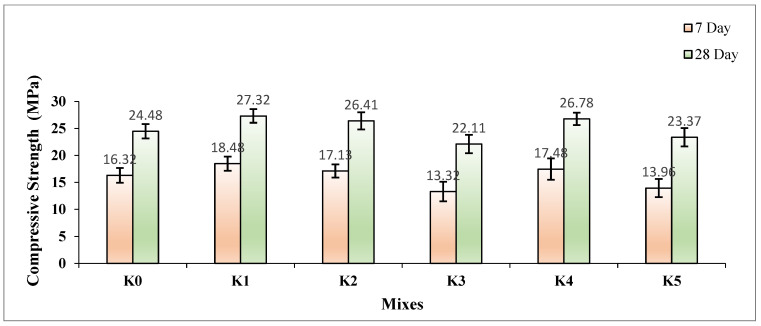
The compressive strength (CS) values of SLWC specimens.

The CS values of the SLWC specimens K0–K5 are provided in Figure 10.

As depicted in Figure 10, the CS values of the SLWC specimens (K0–K5) are presented. At 28 days, the CS value of the control mixture (K0) is 24.48 MPa, which is consistent with the CS value of the mixture, with only the SF addition (K1) being 27.32 MPa. Similarly, the mixtures with 0.3% and 0.6% macro-PP fiber additions (K2 and K3) exhibit CS values of 26.41 and 22.11 MPa, respectively. Furthermore, the CS values of the mixtures (K4 and K5), where micro-PP fibers are used in the hybrid form alongside 0.3% and 0.6% macro-PP fibers, are also 26.78 and 23.37 MPa, respectively. As seen in Figure 9, the results highlight the beneficial influence of SF on both the 7-day and 28-day CS values of the SLWC specimens. Compared to the K0 control sample, the K1 sample with only SF showed an increase in CS values of 13.24% at 7 days and 11.60% at 28 days. This improvement is primarily due to the filler effect of SF, which effectively fills the voids within the SLWC matrix. This process promotes the formation of new calcium silicate hydrate (C-S-H) gels through pozzolanic reactions with hydrated lime (CH), enhancing the overall strength of the concrete. Sounthararajan et al. [64] highlight the beneficial effects of SF on concrete properties, particularly its ability to enhance compressive strength. Xi et al. [65] note that SF’s high silica content and fine particle size make it an effective pozzolanic material. Research by Sounthararajan et al. [64] shows that replacing part of the cement with SF leads to higher compressive strength at both 7 and 28 days compared to standard mixtures. Sounthararajan et al. [64] also suggest that an optimal SF content is 8%, while Zaw [66] identifies 10% as the most effective. Additionally, Sounthararajan et al. [64] emphasize that SF’s micro-filler effect improves the concrete microstructure by filling voids between cement particles. Li [67] has proposed a mathematical model to evaluate concrete strength based on curing time, water–binder ratio, and SF content.

Incorporating micro- and macro-PP fibers into the mixtures can introduce voids within the matrix of the SLWC specimens, which may lead to a decrease in CS values. As the volumetric ratio of PP fibers increases from 0.3% to 0.6%, a more pronounced reduction in the CS values of the SLWC specimens is observed. For instance, the K2 sample, which contains 0.3% macro-PP fibers, exhibited increases in CS values of 4.96% at 7 days and 7.88% at 28 days when compared to the K0 control sample. However, the K3 sample, with a higher fiber content of 0.6% macro-PP fibers, showed decreases in CS values of 18.38% at 7 days and 9.68% at 28 days. This reduction can be attributed to the increased void formation caused by the higher volumetric ratio of PP fibers, which compromises the concrete’s microstructure. On the other hand, the combination of micro-PP fibers with 40 mm long macro-PP fibers effectively addresses this issue by filling the micro-voids within the SLWC matrix. This filling action results in slightly improved compressive strength values for the mixtures. The K4 sample, which includes 0.3% of both micro- and macro-PP fibers, demonstrated increases in CS values of 7.11% at 7 days and 9.40% at 28 days compared to the K0 control sample. Conversely, the K5 sample, with 0.6% micro- and macro-PP fibers, experienced decreases in CS values of 14.46% at 7 days and 4.53% at 28 days. Overall, while higher PP fiber content can lead to greater void formation and reduced CS, the hybrid use of both micro- and macro-PP fibers can mitigate this effect and enhance the performance of the SLWC specimens.

Rasheed et al. [68] and Abbas et al. [69] highlight the varied effects of incorporating PP fibers into concrete mixtures on mechanical properties. Some studies indicate that a low content of PP fibers can enhance CS, while others, such as Partan et al. [70] and Abbas et al. [69], report a decrease in CS at higher fiber percentages. While PP fibers can effectively control crack propagation, Partan et al. [70] note that they may also increase the number of cracks while reducing their depth and length. Overall, Narule and Visapure [71] depicted that PP fibers present a promising option as a partial replacement for steel reinforcement in concrete, enhancing strength and durability.

**Figure 11:**
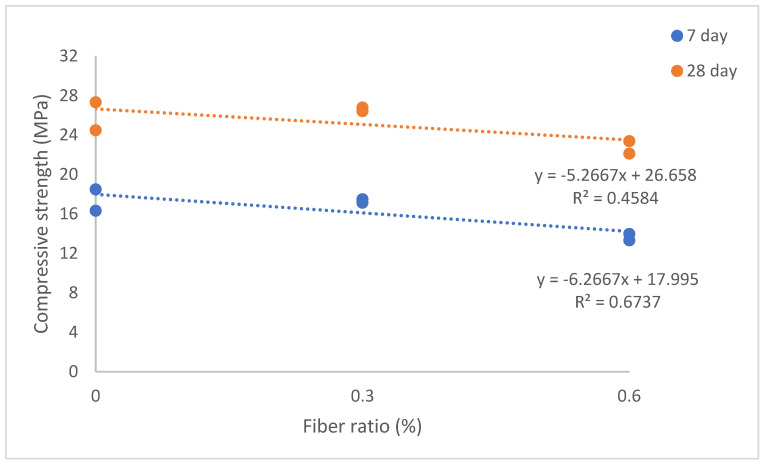
The relationship between fiber volume ratio and compressive strength (CS) values of SLWC specimens.

The analysis of the graph (Figure 11), which illustrates the relationship obtained from the regression analysis between the CS of the samples at 7 and 28 days and the volumetric fiber ratios, reveals that the R^2^ values are notably low. The primary reason for this low correlation is the non-homogeneous integration and distribution of the fibers within the concrete matrix. The distribution of PP fibers may not be uniform throughout the concrete, resulting in inconsistent effects on the CS. This situation underscores the complexity of the fibers’ influence, indicating that their effects are multifactorial and depend on various interrelated factors.

### 4.6. Splitting Tensile Strength of SLWC Specimens

**Figure 12:**
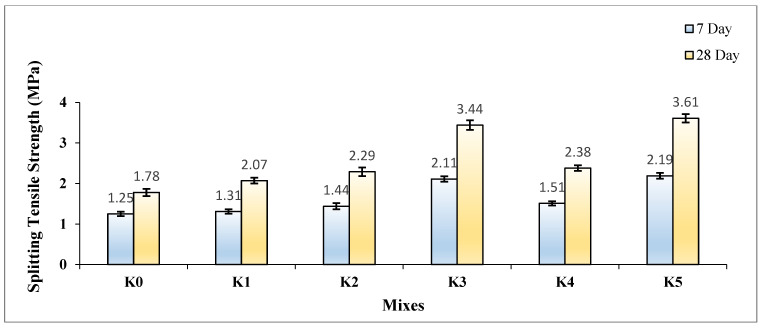
The splitting tensile strength (STS) values of SLWC specimens.

The STS values of the SLWC specimens (K0–K5) are provided in Figure 12.

As depicted in Figure 12, the STS values of the SLWC specimens (K0–K5) exhibit notable variations. At 28 days, the STS value of the control mixture (K0) is recorded at 1.78 MPa, while the mixture with only the SF additive (K1) demonstrates an STS value of 2.07 MPa. Moreover, the mixtures with 0.3% and 0.6% macro-PP fiber additions (K2 and K3) exhibit STS values of 2.29 MPa and 3.44 MPa, respectively. Additionally, the STS values of the mixtures (K4 and K5), where micro-PP fibers are used in the hybrid form alongside 0.3% and 0.6% macro-PP fibers, respectively, are measured at 2.38 MPa and 3.61 MPa. As seen in Figure 12, the results underscore the significant impact of SF on enhancing both the 7-day and 28-day STS values of the SLWC specimens. The K1 sample with only SF demonstrated notable improvements in STS values, showing increases of 4.80% at 7 days and 16.29% at 28 days compared to the K0 control sample. This enhancement is primarily attributed to SF’s filler effect, which effectively fills voids within the SLWC matrix. Additionally, SF promotes the formation of new C-S-H gels through pozzolanic reactions with CH, significantly contributing to the overall strength of the concrete. Shukla and Gupta [72], Gražulytė et al. [73], Zaw [66], and Esmailpour et al. [74] collectively demonstrate the significant impact of SF on enhancing concrete strength properties. Gražulytė et al. [73] indicate that SF improves compressive, tensile, and flexural strengths at both 7 and 28 days. They also report that optimal SF content varies, with 7% being ideal for high-strength concrete, while Zaw [66] found that a 10% replacement yielded the highest CS. Shukla and Gupta [64] and Esmailpour et al. [74] highlight that SF enhances concrete’s durability and resistance to surface cracking. Gražulytė et al. [73] further note that SF improves performance under cyclic loading and enhances the microstructure of concrete. These findings underscore SF’s potential as a valuable supplementary cementitious material for producing high-performance concrete with superior mechanical properties.

Incorporating both micro- and macro-PP fibers into the mixtures effectively mitigates the tensile stresses that occur at the splitting plane of the SLWC specimens, leading to improved STS values. As the volumetric ratio of PP fibers increases from 0.3% to 0.6%, a more substantial enhancement in the STS values is observed. For instance, the K2 sample, which contains 0.3% macro-PP fibers, exhibited increases of 15.20% at 7 days and 28.65% at 28 days compared to the K0 control sample. In contrast, the K3 sample with 0.6% macro-PP fibers showed remarkable increases of 68.80% at 7 days and 93.26% at 28 days. This significant improvement is primarily due to the higher volumetric ratio of PP fibers, which allows more fibers to bridge the tensile cracks that develop during the splitting test. Additionally, the inclusion of 12 mm micro-PP fibers along with 40 mm long macro-PP fibers slightly enhances the increase in STS values for the SLWC specimens. The K4 sample, which includes 0.3% of both types of fibers, showed impressive increases in STS values of 20.80% at 7 days and 33.71% at 28 days compared to the K0 control sample. Conversely, the K5 sample with 0.6% of both micro- and macro-PP fibers achieved even greater increases, showing 75.20% at 7 days and 102.81% at 28 days. This remarkable improvement can be attributed to the ability of the 12 mm long micro-PP fibers to effectively bridge micro-cracks, preventing them from expanding into larger macro-cracks during the splitting test.

Rasheed et al. [68] noted that incorporating 0.4% PP fibers by weight of concrete resulted in the highest compressive strength and a significant increase in STS at 7 days. Liang et al. [75] have shown that incorporating PP fibers into concrete mixtures can significantly enhance mechanical properties, particularly STS. Studies indicate that adding micro- and macro-PP fibers can increase STS by 7–50% compared to plain concrete. Liang et al. [75] also found that an optimal combination of multi-scale PP fibers (6 kg/m^3^ total, with 80% macro-fibers) improved STS by 36% at 800 °C. Abousnina et al. [76] reported that a 6 kg/m^3^ dosage of macro-PP fibers increased STS by 41.9%. These findings demonstrate that PP fibers effectively address tensile stresses in concrete, enhancing its STS and overall performance.

**Figure 13:**
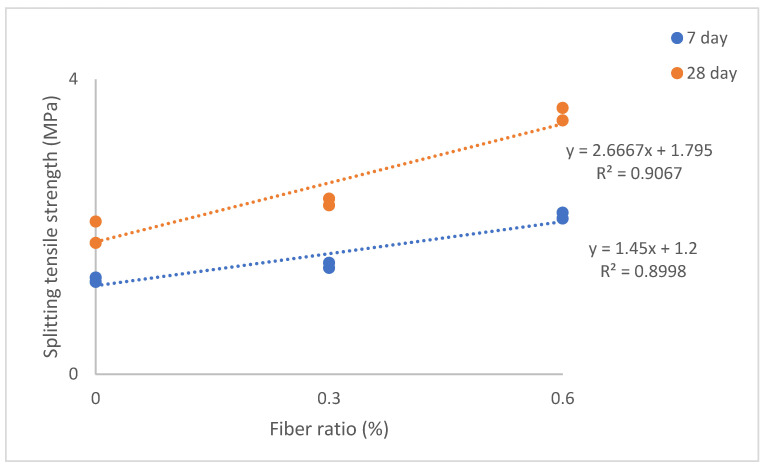
The relationship between fiber volume ratio and splitting tensile strength (STS) values of SLWC specimens.

The analysis in Figure 13 reveals a strong correlation between the STS of the samples at 7 and 28 days and the volumetric fiber ratios, as indicated by the notably high R^2^ values. The primary reason for this strong correlation is the homogeneous integration and distribution of the PP fibers within the concrete matrix. PP fibers demonstrate significant effectiveness in mitigating tensile stresses in the splitting plane of the SLWC samples, resulting in a consistent impact on strength. This situation suggests that the influence of PP fibers is more effective and multifactorial in enhancing the tensile strengths of the SLWC samples.

### 4.7. SEM Analysis of SLWC Specimens

**Figure 14:**
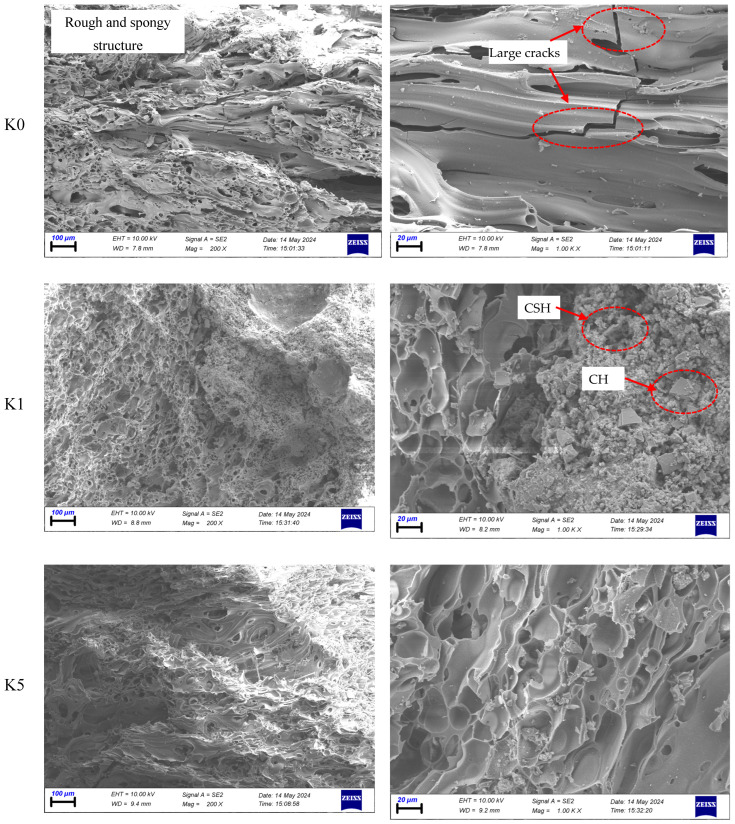
The SEM images of K0, K1, and K5 SLWC specimens.

The SEM images of K0, K1, and K5 specimens is shown in Figure 14. In the SEM images of the control SLWC specimen (K0), the pumice aggregates are observed as irregularly shaped particles, varying in size, and displaying highly porous surfaces. This unique morphology is indicative of their lightweight nature, which contributes to the overall reduction in density of the concrete. The high-resolution images distinctly highlight the porous structure of the pumice aggregates, revealing numerous interconnected voids that enhance the material’s thermal and acoustic insulation properties. Surrounding the pumice aggregates, the concrete matrix is also clearly visible, illustrating the spatial distribution of these aggregates within the cementitious environment. This distribution is critical as it affects the mechanical interlocking and bonding characteristics between the aggregates and the matrix. The SEM analysis provides a detailed view of the interface between the pumice aggregates and the cement matrix, showcasing the nature of this boundary. This interface is crucial for understanding the bonding mechanisms at play, which influence the load transfer and stress distribution within the SLWC matrix. Researchers have conducted scanning electron microscopy (SEM) analyses on lightweight concrete (LWC), revealing crucial insights into the microstructure and properties of these materials. Azhar et al. [77] highlight that pumice aggregates, characterized by their irregular shapes and highly porous surfaces, significantly contribute to the reduced density of LWC. Bonifazi et al. [78] emphasize that the interfacial transition zone (ITZ) between aggregates and cement paste is a vital component affecting concrete properties, with its characteristics varying according to the type of aggregate used. Chung et al. [79] discuss the potential of lightweight aggregates, such as crushed and expanded waste glass, as alternatives to natural aggregates, noting that these materials can influence the pore structure and thermal properties of concrete. Zhang and Gjørv [80] point out that the nature of the ITZ is closely linked to the microstructural characteristics of the aggregate; more porous lightweight aggregates often lead to a denser and more homogeneous interfacial zone, enhancing mechanical interlocking and bonding. These findings underscore the importance of careful aggregate selection in optimizing the performance of LWC. Upon adding SF to the SLWC mixture (K1), significant microstructural changes are observed. The incorporation of SF enhances the bonding between the aggregate particles and the cement paste, attributed to the pozzolanic reaction occurring between the SF particles and calcium hydroxide (CH) present in the concrete. These reactions generate additional C-S-H gel, which is the primary binding phase responsible for the strength and durability of concrete. Researchers have found that the incorporation of SF into concrete mixtures leads to significant microstructural and performance improvements. Sounthararajan and Sivakumar [64] explain that the pozzolanic reaction of SF with CH produces additional C-S-H gel, which is the primary binding phase responsible for the strength and durability of concrete. Justnes [81] notes that SF particles, being much smaller than cement grains, effectively fill voids between them, thereby densifying the concrete matrix. Uzbas and Aydın [82] emphasize that this micro-filling effect enhances the bonding between aggregate particles and the cement paste. Iqbal et al. [83] highlight that this reaction reduces CH content while increasing C-S-H, resulting in a denser microstructure and improved mechanical properties. When micro- and macro-PP fibers are introduced into the SLWC at a volumetric ratio of 0.6% in a hybrid form (K5), the interfacial transition zone between the hybrid PP fibers and the cementitious matrix appears well defined and compact, indicating strong adhesion between the fibers and the surrounding matrix. This robust interface enhances the transfer of stresses between the fibers and the matrix, resulting in improved mechanical properties and durability of the SLWC. Researchers have identified the ITZ between fibers and the cementitious matrix as a crucial factor in the performance of fiber-reinforced concrete. Bajaj et al. [84] explain that engineered PP fibers, when coated with supplementary cementitious materials, can significantly enhance the ITZ. This improvement leads to better bonding between the PP fibers and the matrix, resulting in superior mechanical properties. Silva et al. [85] demonstrate that hybrid polyethylene/PP fibers can provide notable enhancements in mechanical strength and post-cracking ductility in cement-based composites. These hybrid fibers not only improve the initial strength of the concrete but also enhance its ability to deform after cracking, which is essential for maintaining structural integrity under load. In the context of SLWC, Ibrahim and Ibrahim and Abbas [86] highlight that the use of hybrid steel and PP fibers at optimal ratios can substantially increase both flexural and tensile strengths. This combination of fibers effectively improves the concrete’s load-bearing capacity and resistance to deformation. However, Bentur [87] depicted that microstructural changes can occur over time, potentially leading to an enhanced bond and stiffening of the ITZ. This stiffening effect may inadvertently reduce the mechanical properties of the composite, particularly when brittle microfibers are used in dense matrices. Therefore, researchers stress the importance of carefully selecting fiber type, size, and matrix composition to develop durable cementitious composites that exhibit improved mechanical properties and maintain their performance over the long term. This comprehensive approach ensures that the benefits of fiber reinforcement are fully realized while mitigating potential drawbacks associated with the aging and behavior of the composite materials.

## 5. Advantages, Disadvantages, and Recommendations for Researchers

Advantages:

○ Reduced Weight: SLWC significantly decreases the overall weight of structures, leading to lower structural loads.
○ Enhanced Energy Efficiency: The lower thermal conductivity improves energy efficiency in buildings, contributing to reduced heating and cooling costs.
○ Improved Strength Properties: The incorporation of SF enhances CS and STS due to the formation of C-S-H gels. In addition, by incorporating macro-PP fibers thoughtfully into SLWC mixtures, engineers can significantly enhance STS, improving the material’s performance in structural applications.
○ Workability Enhancement: Using a combination of micro-PP and macro-PP fibers improves the workability of SLWC, making it easier to handle and place.
○ Thermal Properties: Macro-PP fibers alone contribute more effectively to improving thermal properties by creating a porous structure.

Disadvantages:

○ Workability Issues with Silica Fume: While SF improves CS and STS, it negatively affects the workability of SLWC mortars.
○ CS Reduction at Higher Fiber Ratios: Increasing the ratios of micro- and macro-PP fibers can lead to lower CS values, which may not be desirable in certain applications.
○ Minor Impact on Thermal Conductivity: The effect of SF on reducing thermal conductivity is limited compared to the benefits of macro-PP fibers.

Recommendations for Field Implementation:

○ Careful Mix Design: Engineers should optimize the proportions of SF and PP fibers to balance workability and strength. It may be beneficial to conduct trial mixturees to determine the ideal formulation for specific applications.
○ Use of Macro-PP Fibers: For projects where thermal performance is critical, it is advisable to prioritize the use of macro-PP fibers due to their effectiveness in enhancing thermal properties and maintaining workability.
○ Monitoring During Placement: Given the workability challenges introduced by SF, it is crucial to monitor the mixing and placement processes closely to ensure proper handling and avoid complications.
○ Application in Lightweight Structures: This material is particularly suited for lightweight construction applications where reduced weight and improved thermal efficiency are key priorities, such as in residential buildings, modular structures, and energy-efficient designs.

## 6. Conclusions

This study investigates the workability, thermal, and mechanical properties of SF and micro- and macro-PP fibers added to SLWC. The study focused on developing new composite material with enhanced eco-friendly, superior strength and durability properties using SF and micro- and macro-PP fibers. Below are the summarized findings.

SF and PP fibers had a notable impact on the workability of SLWC mixtures. Their incorporation led to lower slump values and longer Vebe times, suggesting a decrease in fluidity. This reduced workability may pose challenges during handling and placement, potentially necessitating the use of superplasticizers to achieve the desired performance and ease of application.

Both micro- and macro-PP fibers created voids within SLWC mixtures, which resulted in lower DD and ODD values. This reduction in density can influence the overall mechanical properties and durability of the concrete, potentially impacting its performance in various applications.

SF improved the microstructure of SLWC mixtures, resulting in reduced WA values in the specimens. Conversely, the incorporation of PP fibers increased permeability, leading to higher WA values. This interplay highlights the contrasting effects of SF and PP fibers on the WA properties of SLWC.

PP fibers significantly decreased the thermal conductivity of the SLWC specimens by introducing voids within the mixture. This combination of effects contributes to the overall thermal performance of the concrete, making it more efficient in thermal insulation.

SF improved both CS and STS by effectively filling voids and generating new C-S-H bonds within the matrix. This process leads to a denser, more cohesive structure, ultimately enhancing the mechanical properties of the SLWC specimens.

PP fibers reduced CS in SLWC by introducing voids into the matrix. However, they simultaneously enhanced STS, particularly at higher volume fractions. This improvement occurs because the fibers effectively bridge cracks at the tensile plane, helping to maintain the structural integrity of the concrete under tensile stress.

The hybrid incorporation of micro- and macro-PP fibers enhanced the workability and mechanical properties of the SLWC specimens. However, this combination did not improve thermal conductivity, as the fibers created fewer voids in the matrix, which ultimately affected the overall thermal performance.

Observations show a clear ITZ between the hybrid PP fibers and the cementitious matrix, indicating strong adhesion. This effective bonding improves both the mechanical properties and durability of SLWC, contributing to its overall performance and longevity.

The increasing global waste crisis underscores the importance of integrating waste materials and PP fibers into the production of SLWC for sustainable development. Utilizing waste materials not only reduces landfill contributions but also enhances the mechanical properties of concrete, leading to improved performance. Incorporating PP fibers further contributes to the durability and ductility of SLWC, addressing tensile stresses effectively. Thus, the adoption of these sustainable practices in concrete production is crucial for minimizing environmental impact while meeting the rising demands of modern infrastructure development.

SF significantly improved the microstructure and overall strength of SLWC by filling voids and forming new bonds within the matrix. This enhancement led to better mechanical performance and reduced water absorption. On the other hand, PP fibers, while introducing voids that decreased compressive strength, played a crucial role in enhancing crack-bridging capabilities. This means that, under tensile stress, the fibers helped maintain the integrity of the concrete by preventing crack propagation. The hybrid use of both micro- and macro-PP fibers yielded promising results, notably improving the workability and mechanical properties of the SLWC. This combination indicates potential pathways for further optimizing SLWC formulations to achieve a balance among strength, durability, and ease of application. Overall, these findings underscore the importance of carefully selecting and combining materials to enhance the performance of lightweight concrete systems.

In summary, this study focused on the effects of SF and PP fibers on the workability, thermal, and mechanical properties of SLWC. While SF significantly enhanced the microstructure and strength, the study lacked a long-term performance analysis, particularly regarding water absorption and chemical resistance. The impact of varying PP fiber volume fractions was not thoroughly explored, which could help identify the optimal amount for improving mechanical properties. Additionally, the potential benefits of incorporating other additives alongside SF and PP fibers were not considered, limiting overall performance enhancement. The microstructural analysis of the interactions among SF, PP fibers, and the cementitious matrix was insufficient. Conducting an SEM analysis on a larger number of specimens could provide deeper insights into these relationships. Future research should include long-term durability tests to assess how SLWC performs under different environmental conditions. Investigating various combinations of SF and PP fibers, along with other additives, could lead to more effective formulations. Furthermore, real-world applications and performance monitoring are crucial for validating laboratory findings. Finally, ecological, and economic assessments of SF and PP fibers should be integrated into future studies to promote sustainable practices. Overall, these recommendations emphasize the importance of both SF and PP fibers in enhancing SLWC performance.

## Figures and Tables

**Table 1:** The chemical properties of PA_s_.

Component	Weight (%)
SiO_2_	75.4
Al_2_O_3_	14.6
Fe_2_O_3_	2.37
Na_2_O	0.22
MgO	0.04
K_2_O	5.24
CaO	1.30
TiO_2_	0.53
Loss on ignition (LOI)	3.30

**Table 2:** The physical properties of PA_s_.

Aggregate Size (mm)	0–4	4–8	8–16
Water absorption (%)	36.72	38.13	40.13
Bulk density (kg/m^3^)	458	382	347

**Table 3:** The chemical and physical properties of PC and SF.

Chemical Properties	PC	SF
(Component)	Weight (%)	Weight (%)
SiO_2_	60.34	2.44
Al_2_O_3_	23.89	1.79
Fe_2_O_3_	7.59	91.41
CaO	2.01	0.31
MgO	2.04	0.06
Na_2_O	0.27	1.95
K_2_O	0.39	0.22
Loss on ignition (LOI)	2.43	1.87
Density(g/cm^3^)	3.13	2.18
Specific surface area (cm^2^/g)	3491	18,650

**Table 4:** The technical properties of micro- and macro-PP fibers.

	Length (mm)	Diameter	Aspect Ratio (L/d)	Density (g/cm^3^)	Tensile Strength (MPa)	Elastic Modulus (MPa)
12 mm micro-PP fibers	12	18–20 μm	-	0.91	500–700	2000–2800
40 mm macro-PP fibers	40	0.72 mm	56	0.91	550	3500

**Table 5:** The mixture design of the SLWC mixtures (kg/m^3^).

Mixtures	Mixture Code	Cement	SF	(0–4 mm) (PA)	(4–8 mm) (PA)	(8–16 mm) (PA)	Water	SP (%)	*w*/*c*
Control	K0	400	-	480	200	170	208	1	0.52
SF10%	K1	360	40	480	200	170	208	1	0.52
SF10%PP40-0.3%	K2	360	40	480	200	170	208	1	0.52
SF10%PP40-0.6%	K3	360	40	480	200	170	208	1	0.52
SF10%PP40-0.2%-PP12-0.1%	K4	360	40	480	200	170	208	1	0.52
SF10%PP40-0.4%-PP12-0.2%	K5	360	40	480	200	170	208	1	0.52

## Data Availability

The original contributions presented in the study are included in the article, further inquiries can be directed to the corresponding authors.
